# Long-term dietary supplementation with natural honey does not predispose growing male rats to metabolic syndrome

**DOI:** 10.1186/1753-6561-6-S3-P3

**Published:** 2012-06-01

**Authors:** Abdulwahid Ajibola, Joseph P Chamunorwa, Kennedy H Erlwanger

**Affiliations:** 1 Department of Physiology, Faculty of Basic Medical Sciences, Olabisi Onabanjo University, Ikenne 121002, Ogun State, Nigeria; 2 School of Physiology, Faculty of Health Sciences, University of the Witwatersrand, 7 York Road, Parktown 2193, South Africa; 3 Department of Veterinary Anatomy and Physiology, Faculty of Veterinary Science, University of Pretoria, Onderstepoort, South Africa

## Background

Metabolic syndrome (MetS) is a condition characterized by abdominal obesity, hyperglycaemia, hypertension and dyslipidaemia, and thus increased susceptibility to diabetes, kidney and heart diseases [1]. The modern human diet contains refined sugars mainly fructose, which is culpable in the global incidence and prevalence of MetS in adults and children [2,3]. We investigated the effects of two dietary sources of fructose, natural honey (NH) and golden syrup (GS) on metabolism in growing animal models (Sprague Dawley rats) fed from neonatal age.

## Methodology

Thirty suckling (7-day old male and female) rats were fed either NH or GS at low (10ml kg^-1^ b.wt) or high (20ml kg^-1^ b.wt) dose daily via stomach tube for 14 days, while control group was gavaged with distilled water. The rats divided into five groups (n= 6) nursed freely between gavages. On weaning, NH or GS was mixed with rat chow as low (20%) or high, 50% (volume/weight, v/m), while 20% (v/w) tap water was added to the control diet. The rats were subjected to an oral glucose tolerance test (OGTT) 48hours before the end of the 13-week study. Thereafter, the rats were euthanazed at term (age -13 weeks old) for visceral measurements; and blood levels of metabolic substrates, leptin, insulin, and hepatic storage of glycogen and lipids were also obtained.

## Results

NH significantly (p<0.01) increased total body weight (TBW), than the other diets. GS increased circulating fasting glucose, triglycerides and free fatty acids (p<0.05). Contrary to the GS-fed rats, the NH-fed animals showed tolerance to an oral glucose load. Golden syrup also significantly increased (p<0.001) visceral fat weight, and caused hepatomegaly unlike NH. There were also hypercholesterolemia, hyperinsulinemia and increased hepatic fat stores in the GS-fed rats relative to NH groups**.** The difference in the hepatic glycogen content between the GS and NH fed rats did not attain any significance at both doses. The area under the curves (AUC) calculated from the OGTT results showed the tendency of our GS-fed rats to hyperglycemia, while the NH-fed rats were normoglycemic. The increased levels of metabolic substrates and visceral fat weight were not observed in the NH-fed rats.

## Conclusion

NH induced healthy growth in rats as previously observed [4], due to its several micronutrients, antioxidants and phytochemical constituents [5,6]. The rats fed GS diets for 13 weeks were predisposed to developing diet-induced MetS. Unlike GS, feeding rats NH from an early age did not cause susceptibility to MetS. NH is a healthy source of dietary sugars, as the rats were not predisposed to dietary-induced MetS [7]. This suggested potential nutritional benefits of substituting honey for refined sugars in animal feed and by extension human diet.
